# On-treatment blood TMB as predictors for camrelizumab plus chemotherapy in advanced lung squamous cell carcinoma: biomarker analysis of a phase III trial

**DOI:** 10.1186/s12943-021-01479-4

**Published:** 2022-01-03

**Authors:** Tao Jiang, Jianhua Chen, Xingxiang Xu, Ying Cheng, Gongyan Chen, Yueyin Pan, Yong Fang, Qiming Wang, Yunchao Huang, Wenxiu Yao, Rui Wang, Xingya Li, Wei Zhang, Yanjun Zhang, Sheng Hu, Renhua Guo, Jianhua Shi, Zhiwu Wang, Peiguo Cao, Donglin Wang, Jian Fang, Hui Luo, Yi Geng, Chunyan Xing, Dongqing Lv, Yiping Zhang, Junyan Yu, Shundong Cang, Yaxi Zhang, Jiao Zhang, Zeyu Yang, Wei Shi, Jianjun Zou, Caicun Zhou, Shengxiang Ren

**Affiliations:** 1 Department of Medical Oncology, Shanghai Pulmonary Hospital, Tongji University School of Medicine, No.507, Zhengmin Road, Shanghai, 200433 China; 2 Hunan Cancer Hospital, Changsha, China; 3 Northern Jiangsu People’s Hospital, Yangzhou, China; 4 Jilin Cancer Hospital, Changchun, China; 5 Harbin Medical University Cancer Hospital, Harbin, China; 6 Anhui Provincial Hospital, Hefei, China; 7 Sir Run Run Shaw Hospital Zhejiang University School of Medicine, Hangzhou, China; 8 Henan Cancer Hospital, Zhengzhou, China; 9 Yunnan Cancer Hospital & The Third Affiliated Hospital of Kunming Medical University & Yunnan Cancer Centre, Kunming, China; 10 Sichuan Provincial Cancer Hospital, Chengdu, China; 11 Anhui Chest Hospital, Hefei, China; 12 The First Affiliated Hospital of Zhengzhou University, Zhengzhou, China; 13 The First Affiliated Hospital of Nanchang University, Nanchang, China; 14 Shaanxi Provincial Cancer Hospital, Xi’an, China; 15 Hubei Cancer Hospital, Wuhan, China; 16 Jiangsu Province Hospital, Nanjing, China; 17 LinYi Cancer Hospital, Linyi, China; 18 Tangshan People’s Hospital, Tangshan, China; 19 The Third Xiangya Hospital of Central South University, Changsha, China; 20 Chongqing University Cancer Hospital, Chongqing, China; 21 Beijing Cancer Hospital, Beijing, China; 22 Jiangxi Cancer Hospital, Nanchang, China; 23 Baoji Central Hospital, Baoji, China; 24 Jinan Central Hospital, Jinan, China; 25 Taizhou Hospital of Zhejiang Province, Taizhou, China; 26 Zhejiang Cancer Hospital, Hangzhou, China; 27 Heping Hospital Affiliated to Changzhi Medical College, Changzhi, China; 28 Henan Provincial People’s Hospital, Zhengzhou, China; 29 Genecast Biotechnology Co., Ltd., Wuxi City, China; 30 Jiangsu Hengrui Pharmaceuticals Co., Ltd., Shanghai, China

**Keywords:** immunotherapy, PD-1, lung squamous cell carcinoma, blood tumor mutational burden, biomarker

## Abstract

**Background:**

Camrelizumab plus chemotherapy significantly prolonged progression-free survival (PFS) and overall survival (OS) compared to chemotherapy alone as first-line treatment in advanced lung squamous cell carcinoma (LUSC) in the phase III trial (CameL-sq), which has become an option of standard-of-cares for Chinese patients with advanced LUSC. However, the predictive biomarkers remain unknown.

**Methods:**

Tumor tissue samples at baseline, and peripheral blood samples at baseline (pretreatment) and after two cycles of treatment (on-treatment) were prospectively collected from 270 LUSC patients from the CameL-sq study. Blood tumor mutation burden (bTMB) and its dynamics were analyzed to explore their predictive values.

**Results:**

Pretreatment bTMB was not associated with objective response, PFS and OS in camrelizumab or placebo plus chemotherapy groups. Low on-treatment bTMB was associated with significantly better objective response (73.8% vs 27.8%, *P* < 0.001), PFS (median, 9.1 vs 4.1 months; *P* < 0.001) and OS (median, not reached vs 8.0 months; *P* < 0.001) in camrelizumab plus chemotherapy group whereas it did not correlate with objective response and PFS in chemotherapy alone group. Importantly, on-treatment bTMB level could discriminate patients of initially radiological stable disease who would long-term benefit from camrelizumab plus chemotherapy (low vs high, median OS, 18.2 vs 7.8 months; *P* = 0.001). Combing on-treatment bTMB and its dynamics improved the ability for predicting the efficacy of camrelizumab plus chemotherapy.

**Conclusion:**

On-treatment bTMB together with its dynamics could serve as a predictive biomarker for camrelizumab plus chemotherapy in patients with advanced LUSC.

**Trial registration:**

ClinicalTrials.gov identifier: NCT03668496.

**Supplementary Information:**

The online version contains supplementary material available at 10.1186/s12943-021-01479-4.

## Background

Lung squamous cell carcinoma (LUSC) accounts for 25%-30% of non-small-cell lung cancer (NSCLC) [1]. It remains a big challenge to manage the advanced LUSC due to the rare established actionable genomic targets [2, 3]. To date, immunotherapy targeting immune checkpoint inhibitors (ICIs) has significantly revolutionized the treatment landscape of advanced NSCLC [4, 5]. Several studies have demonstrated that ICIs plus chemotherapy could dramatically prolong progression-free survival (PFS) and/or overall survival (OS) in patients with advanced LUSC irrespective of programmed death-ligand 1 (PD-L1) status [6–9]. However, the predictive biomarkers for this combination regimen remain largely unknown.

Tumor mutation burden (TMB) serves as a candidate biomarker for the efficacy of ICI monotherapy in various solid tumors [10–15]. However, a substantial proportion of patients could not provide sufficient tissue for TMB calculation using next generation sequencing (NGS). Given the convenience and non-invasiveness, TMB calculated using peripheral blood circulating tumor DNA (ctDNA), named blood TMB (bTMB), is becoming an attractive approach [16]. Besides that, bTMB calculating from ctDNA could attenuated the potential sampling biases due to intra-tumoral heterogeneity or low tumor content. It also provides the possibility to longitudinally collect the peripheral blood samples for dynamically monitoring the early on-treatment changes. Meanwhile, several disadvantages also existed. For example, the minimum amount of ctDNA, the panel size and various variants that should be included for bTMB calling, the cutoff and time point to assess bTMB remained undetermined, leaving questions about the optimal approach and difficulty of results integration. Nevertheless, two elegant proof-of-concept studies have revealed that bTMB could robustly identify patients who could derive clinically significant improvements in treatment outcomes from ICI monotherapy [17, 18]. However, the robustness of its predictive value for immunotherapy plus chemotherapy still warrants prospective investigations.

CameL-sq is randomized, double-blind, phase III trial conducted in 53 medical centers in China (ClinicalTrials.gov identifier: NCT03668496) to evaluate the efficacy and safety of camrelizumab (a humanized IgG4-κ monoclonal antibody against PD-1) plus chemotherapy as first-line treatment for patients with advanced LUSC. The results showed that camrelizumab plus chemotherapy significantly improved PFS and OS compared to placebo plus chemotherapy as first-line treatment in advanced LUSC [19]. This study also prospectively exploring the predictive value of bTMB and its dynamics for advanced LUSC treated with camrelizumab plus chemotherapy, peripheral blood samples were collected at the beginning of initial treatment (pretreatment) and after two cycles of treatment (on-treatment). Here, we reported the final results of biomarker analyses.

## Materials and methods

### Study design

Patients with previously untreated, pathologically confirmed stage IIIB-IV LUSC without sensitizing EGFR or ALK genomic aberration were randomized (1:1) to receive camrelizumab (200 mg) or placebo in combination with carboplatin (area under the curve 5 mg/mL*min) plus paclitaxel (175 mg/m^2^) for 4-6 cycles at investigator’s discretion, followed by maintenance therapy with camrelizumab or placebo until disease progression (PD), or intolerable toxicity. The stratification factors were smoking history (≥400 cigarettes-year vs <400 cigarettes-year vs never), presence of liver or brain metastases (both sites vs one site vs none), and sex (male vs female). Patients in the placebo plus chemotherapy group with independent review committee (IRC)-assessed PD were allowed to cross over to receive camrelizumab monotherapy. The maximum exposure during of camrelizumab was two years. The primary endpoint was IRC-assessed PFS, defined as time from randomization to the first RECIST version 1.1-defined PD or death from any cause, whichever occurred first. OS and investigator-assessed PFS, objective response rate (ORR), disease control rate (DCR) and duration of response were prespecified secondary endpoints. Complete response (CR), partial response (PR) or stable disease (SD) was required to be confirmed with a subsequent scan at least four weeks after the initial documentation. Survival was followed up every three months after treatment discontinuation. PD-L1 tumor proportion score (TPS) was centrally assessed by using a PD-L1 immunohistochemistry kit (E1L3N, AmoyDx, Xiamen, China). The clinical protocol was approved by the respective institutional review boards and ethics committees. All participants provided written informed consent.

### Sample collection

Fresh (from core needle biopsy) or formalin-fixed paraffin-embedded (FFPE) baseline samples were collected before the protocol-defined treatments. Fresh biopsy samples were snap-frozen in liquid nitrogen within 30 minutes. Pretreatment and on-treatment peripheral blood samples (10 mL, EDTA tubes) were collected.

### DNA extraction and library preparation

DNA was extracted from tumor tissues using GeneRead DNA FFPE Kit (Qiagen 180134, Hilden, Germany) and from peripheral blood lymphocyte with TGuide S32 Magnetic Blood Genomic DNA Kit (TIANGEN DP601-T5C China) according to the manufacturer’s recommended protocol. Cell free DNA (cfDNA) was extracted using MagMAX Cell-Free DNA Isolation kit (ThermoFisher, A29319 USA). DNA samples were quantified with the Qubit dsDNA HS Assay kit (Life Technologies, Q32854 USA) following the manufacturer’s instructions. Genomic DNA was sheared into 150-200 base pairs (bp) fragments through Covaris LE220 using the recommended settings for NGS library preparation. KAPA Hyper Prep Kit (KAPA Biosystems, Massachusetts, KK8504 USA) was used for fragmented DNAs construction according to the manufacturer’s instruction. All fragmented gDNA or cfDNA (10 to 50 ng) was end-repaired, sequencing adaptor-ligated, and PCR amplified before purification with 0.8X Agencourt AMPure XP beads (Beckman Coulter, Brea, CA, A63882 USA). The concentration and quality of the library was determined using the Qubit 3.0 system and Bioanalyzer 2100 (Agilent, Agilent HS DNA Kit, 5067-4626).

### Genomic sequencing

A fraction of each library was hybridized to a predesigned panel covering 1.6 Megabase of genomic regions covering 543 cancer-related genes using HyperCap Target Enrichment Kit (Roche, 8286345001 Swiss). After hybridization and washing according to the manufacturer’s protocol, the KAPA HiFi HotStart ReadyMix (KAPA Biosystems, Massachusetts, KK2602 USA) was used for the amplification of captured libraries. Then, the capture libraries were purified with 1X AMPure, quantified, and pooled for sequencing on Illumina Novaseq 6000 with Paired end 150 bp mode.

### Bioinformatic pipeline

After filtering the low quality reads by Trimmomatic version 0.36 [20], clean reads were aligned to the human reference genome (hg19, NCBI Build 37.5) with the Burrows-Wheeler Aligner version 0.7.17. The Picard toolkit version 2.23.0 [21] was utilized for sorting, making duplicates. Realignment was done using Genome Analysis ToolKit version 3.7 [22] and VarDict version 1.5.1 [23] was applied to call single nucleotide variation (SNV) mutations while complex heterozygous mutations were merged by FreeBayes version 1.2.0. ANNOVAR software too l [24] was used to annotate the mutations. Typical QC-filtering such as variant quality and strand bias was used to the raw variant list. Additionally, variants in low complexity repeat and segmental duplication regions that matched to the low mappable regions defined by ENCODE [25], as well as variants in an internally developed and validated list of recurrent sequence-specific errors were removed. After removing germline or hematopoietic origin mutations using paired normal sample, somatic mutations met the following criterions were used for the following analysis: (i) the sequencing depth was more than 100X for tissue samples and 500X for plasma samples; (ii) the variant allele frequency (VAF) threshold of SNV was 4% and that of insertions/deletions (InDels) was 5%. These quality cut-offs were predetermined during the analytical validation of the internal NGS panel to optimize the test performance and measure according to sensitivity, specificity, repeatability and reproducibility.

### Definition of bTMB and its dynamics

Nonsynonymous mutations (including SNVs and InDELs) in the coding regions were selected for the following analysis of bTMB, while driver gene mutations and hotspot mutations included in the dbSNP138/COSMIC database were removed. Mutations that met a certain sequencing depth (100X for tissue samples and 500X for plasma samples) and VAF (5% for tissue samples and 0.7% for plasma samples) were chose as candidate mutations for bTMB analysis. Subsequently, bTMB was calculated based on the candidate mutations according to the following formula:

$$bTMB=\frac{Absolute\ Mutaion\ Count\ast 1000000}{Panel\ exonic\ base\ num}.$$

The on-treatment bTMB for each patient was defined as the bTMB detected at the on-treatment timepoint. The ∆bTMB (bTMB dynamics) was calculated per patient as the on-treatment bTMB level minus the pretreatment bTMB level.

### Statistical analysis

Correlations between high and low bTMB groups were analyzed using the chi-squared or Fisher’s exact test for categorical variables. The continuous variables were analyzed by ANOVA and Tukey’s multiple comparison tests. Mann-Whitney U tests or Kruskal-Wallis rank sum tests were used for comparisons of continuous variables across multiple groups. The Kaplan-Meier curves were used to estimate the median survival time of PFS and OS, with the 95% CIs estimated using the Brookmeyer and Crowley method. Between-group comparison in PFS and OS were assessed using a stratified log-rank test. Hazard ratios (HRs) and associated 95% confidence intervals (CIs) were calculated based on a stratified Cox proportional-hazards model. ORR and DCR were analyzed and the corresponding 95% CIs were estimated using the Clopper-Pearson method; between-group comparisons were assessed using the stratified Cochran-Mantel-Haenszel method. All statistical analyses were conducted using GraphPad PRISM 6.0 and the SPSS statistical software, version 22.0 (SPSS Inc., Chicago, IL, USA). Two-side *P* < 0.05 was considered statistically significant.

## Results

### Patient characteristics

**Fig. 1:**
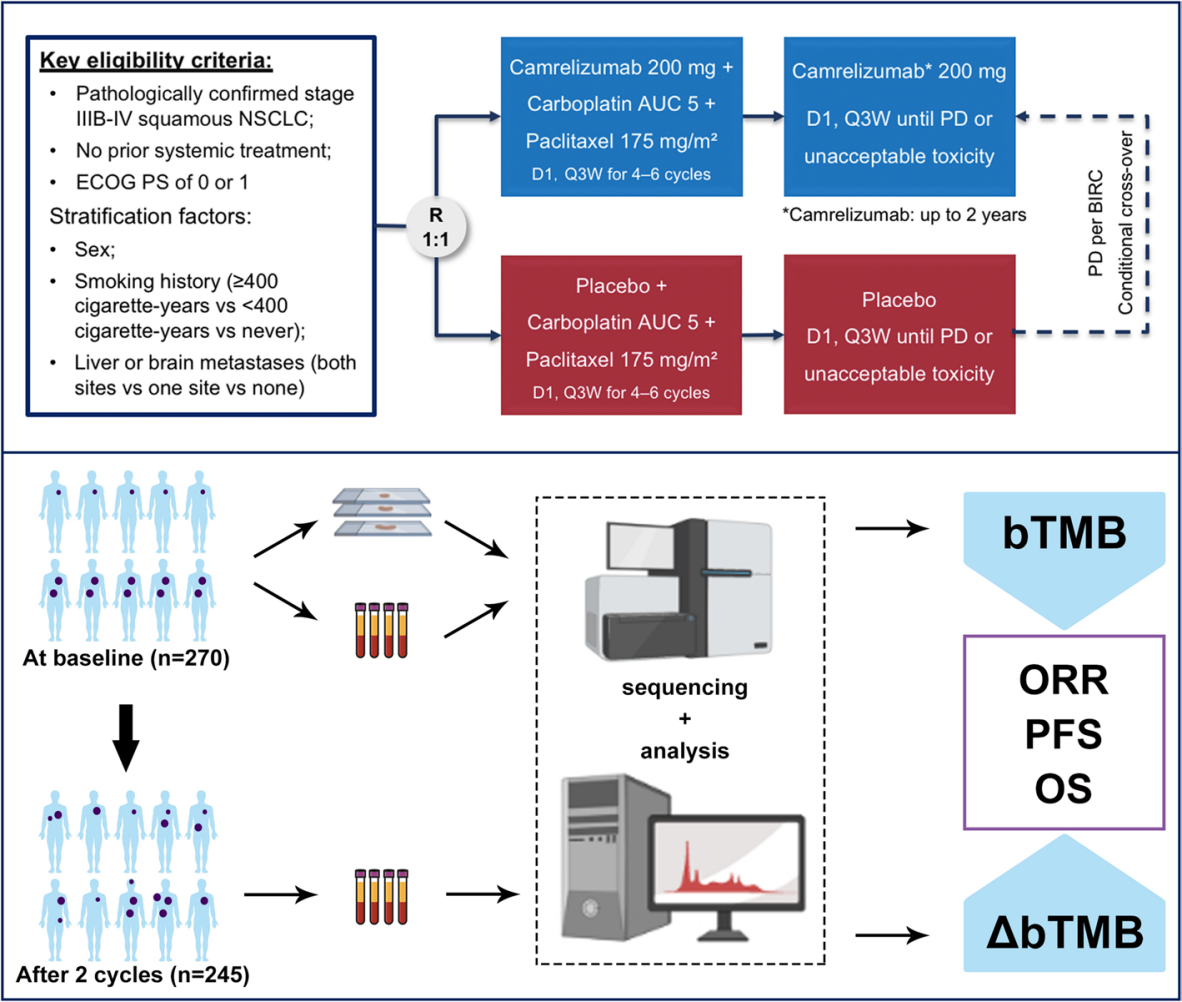
Study design

A total of 389 patients with previously untreated, stage IIIB-IV LUSC were enrolled and randomized to receive camrelizumab plus chemotherapy (N=193) or placebo plus chemotherapy (N=196). In total, pretreatment tissue and blood samples from 270 patients (134 from camrelizumab plus chemotherapy and 136 from placebo plus chemotherapy group) were collected for this biomarker analysis (Figure 1). Baseline clinical features including age, sex, smoking history, Eastern Cooperative Oncology Group performance status (ECOG PS), disease stage, liver and/or brain metastasis, and PD-L1 expression as well as PFS and OS were similar between the biomarker evaluable cohort (BEC) and intention-to-treat (ITT) population (Table S1 and Figure S1). Therefore, we assumed that this biomarker analysis of BEC could represent the ITT population in this study. Major clinical parameters were balanced between camrelizumab and placebo plus chemotherapy groups (Table S1).

### Detectability of ctDNA, bTMB and tissue TMB

Among the BEC, all of the included pretreatment tissue samples were qualified and could be calculated tissue TMB (tTMB). ctDNA and bTMB were detected in the pretreatment samples of 132 of 134 (98.5%) in camrelizumab plus chemotherapy group and 135 of 136 (99.3%) in chemotherapy group. The median tTMB was 7.6 Mutations/Megabase (Muts/Mb, range 1.3–42.3) in camrelizumab plus chemotherapy group and 7.8 Muts/Mb (range 1.3–33.0) in chemotherapy group. The median bTMB was 7.7 Muts/Mb (range 0.0–47.3) in camrelizumab plus chemotherapy group and 6.4 Muts/Mb (range 0.0–36.1) in chemotherapy group. The baseline bTMB correlated well with the matched tTMB (Figure S2A) and patients with ECOG PS of 1 had lower bTMB than those with ECOG PS of 0 (*P* = 0.019, Figure S2H). Whereas baseline bTMB did not correlate with baseline ctDNA concentration, the sum of the diameters of the target lesions, age, sex, smoking history, disease stage, number of distant metastases, liver and brain metastasis, and PD-L1 expression (Figure S2).

### Predictive and prognostic value of pretreatment bTMB and tTMB

**Fig. 2:**
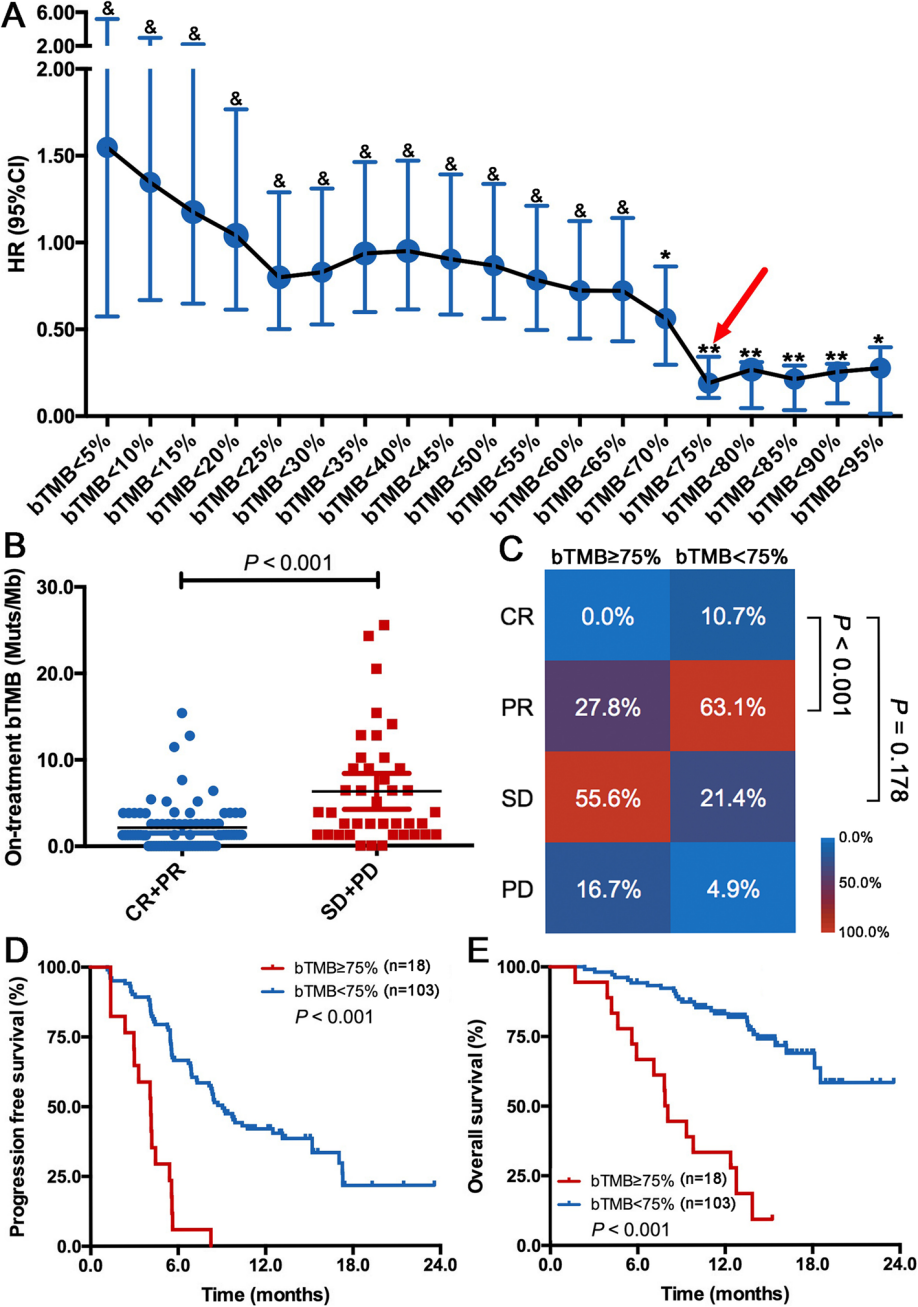
On-treatment bTMB is predictive of immunotherapy plus chemotherapy benefit.** (A)** Forrest plot of hazard ratio (HR) and 95% confidence interval (CI) of PFS by using different on-treatment bTMB level as the cutoff. **(B)** Patients with CR/PR had a significantly lower on-treatment bTMB than those with SD/PD in camrelizumab plus chemotherapy group. **(C)** ORR was significantly higher in patients with low on-treatment bTMB than those with high on-treatment bTMB in camrelizumab plus chemotherapy group. Lower on-treatment bTMB was associated with significantly longer PFS **(D)** and OS **(E)** than those with higher on-treatment bTMB. &, *P* > 0.05; *, *P* < 0.05; **, *P* < 0.01

To investigate the predictive and prognostic value of pretreatment bTMB and tTMB, we defined the high-bTMB or tTMB group as that with a TMB value ≥75% level and the low-bTMB/tTMB group as that with a TMB value below 75% level. Although this definition for TMB cutoff is not popular in the research setting, it is more helpful for us to clarify the relevant investigations due to its briefness and shows the optimal predictive significance (Figure 2A). As shown in Figure S3, both pretreatment tTMB and bTMB did not correlated with objective response rate (ORR), PFS and OS in camrelizumab plus chemotherapy group. Intriguingly, patients obtained complete/partial response (CR/PR) had markedly higher pretreatment tTMB than those with stable disease/disease progression (SD/PD) in chemotherapy group (*P* = 0.032, Figure S4A). Furthermore, a significant association was found between pretreatment tTMB and PFS (*P* < 0.001, Figure S4B) and OS (*P* < 0.001, Figure S4C) in chemotherapy group. Whereas pretreatment bTMB did not correlate with ORR, PFS and OS in this group (Figure S4D-E). Taken together, the lack of association with ORR and treatment outcomes suggests that pretreatment tTMB and bTMB levels may not be prognostic and predictive of response to camrelizumab plus chemotherapy.

### On-treatment bTMB is predictive of immunotherapy plus chemotherapy benefit

Previous studies have reported that on-treatment ctDNA and its dynamics was associated with response and survival in various solid tumors (e.g. melanoma, NSCLC, urothelial cancer, and etc.) treated with ICI monotherapy [26–30]. Whether on-treatment bTMB was predictive and/or prognostic for immunotherapy plus chemotherapy remained unknown. Herein, we analyzed paired on-treatment ctDNA samples of 121 patients from camrelizumab plus chemotherapy group (Fig. 1 and Table 1). In contrast to pretreatment bTMB, high on-treatment bTMB was associated with age≥65 years old (*P* = 0.010, Figure S5A), ECOG PS of 1 (*P* = 0.005, Figure S5D) and ≥3 distant metastases (*P* = 0.031, Figure S5F). Of note, patients with high on-treatment bTMB had marginally statistically longer sum of the diameters of the target lesions after two cycles treatment (*P* = 0.063, Figure S6A). On-treatment bTMB level also correlated with the sum of the diameters of the target lesions after two cycles treatment (*r*^*Spearman*^= 0.327, *P* = 0.009, Figure S6B). Importantly, patients with CR/PR had a significantly lower level of on-treatment bTMB than those with SD/PD (*P* < 0.001, Figure 2B) in camrelizumab group. The ORR was significantly higher in patients with low on-treatment bTMB received camrelizumab plus chemotherapy than those with high on-treatment bTMB (73.8% vs 27.8%, *P* < 0.001; Figure 2C). Moreover, low on-treatment bTMB was associated with dramatically longer PFS (median, 9.1 vs 4.1 months; HR = 0.190, *P* < 0.001; Figure 2D) and OS (median, not reached vs 8.0 months; HR = 0.144, *P* < 0.001; Figure 2E) than those with high on-treatment bTMB. The significant associations with PFS (adjusted HR = 0.189; 95%, 0.101–0.358; *P* < 0.001) and OS (adjusted HR = 0.152; 95% CI, 0.075–0.308; *P* < 0.001) remained after the adjustments of clinical characteristics, pretreatment tTMB and bTMB (Table 2). Conversely, on-treatment bTMB did not correlate with ORR and PFS in chemotherapy group (Figure S7).

**Table 1:** Baseline characteristics of included patients at baseline and after two cycles treatment.

	Patients enrolled at baseline	Patients enrolled after two cycles treatment
	**(*n*=134)**	**(*n*=121)**
Age		
Median (range), years	64 (34-74)	64 (34-74)
≥65 years	79 (59%)	67 (55%)
<65 years	55 (41%)	54 (45%)
Sex		
Male	128 (96%)	116 (96%)
Female	6 (4%)	5 (4%)
Smoking history		
≥400 cigarette-years	117 (87%)	107 (88%)
<400 cigarette-years	6 (4%)	5 (4%)
Never	11 (9%)	9 (8%)
ECOG performance status		
0	25 (19%)	21 (17%)
1	109 (81%)	100 (83%)
Disease stage		
IIIB/IIIC	40 (30%)	34 (28%)
IV	94 (70%)	87 (72%)
Liver or brain metastases at enrollment^*^	0 (0%)	0 (0%)
Liver metastases	14 (10%)	14 (12%)
Brain metastases	2 (1%)	2 (2%)
PD-L1 tumor proportion score		
<1%	61 (46%)	57 (47%)
≥1%	70 (52%)	61 (50%)
1-49%	36 (27%)	37 (31%)
≥50%	34 (25%)	24 (20%)
Not evaluable	3 (2%)	3 (2%)

Data are n (%), unless otherwise indicated. ^*^ No patients with both liver and lung metastases were enrolled. ECOG, Eastern Cooperative Oncology Group

**Table 2:** Univariate and multivariate analyses of clinical parameters on clinical outcomes

Factors	Univariate analysis			Multivariate analysis		
	HR (log rank)	95% CI	*P* value	HR (log rank)	95% CI	*P* value
***Progression-free survival***						
Sex (Female/male)	1.124	0.727-1.737	0.599			
Age (≥65/<65)	0.990	0.312-3.140	0.986			
Smoking (yes/no)	0.825	0.412-1.651	0.587			
ECOG PS (1/0)	1.296	0.716-2.348	0.392			
Stage (IV/III)	1.577	0.943-2.636	0.082	1.415	0.797-2.511	0.236
PD-L1 expression (<1/>1)	1.570	1.011-2.439	0.045	1.604	1.033-2.493	0.035
Number of metastases (>3/<3)	1.580	1.0168-2.458	0.042	1.204	0.596-1.759	0.933
Liver metastasis (yes/no)	1.545	0.769-3.107	0.222			
Brain metastasis (yes/no)	0.772	0.107-5.577	0.798			
Sum of diameters (<median/>median)	0.941	0.609-1.453	0.784			
Tissue TMB at baseline (<75%/>75%)	1.483	0.982-2.255	0.064	1.574	0.993-2.470	0.084
Blood TMB at baseline (<75%/>75%)	1.186	0.781-1.820	0.421			
On-treatment blood TMB (<75%/>75%)	0.190	0.105-0.342	<0.001	0.189	0.101-0.358	<0.001
***Overall survival***						
Sex (Female/male)	1.254	0.301-5.223	0.755			
Age (≥65/<65)	1.489	0.812-2.729	0.198			
Smoking (yes/no)	0.777	0.277-2.180	0.632			
ECOG PS (1/0)	1.184	0.525-2.670	0.683			
Stage (IV/III)	1.106	0.564-2.169	0.770			
PD-L1 expression (<1/>1)	1.893	1.010-3.547	0.046	2.175	1.154-4.101	0.016
Number of metastases (<3/>3)	0.525	0.286-0.962	0.037	0.665	0.355-1.245	0.202
Liver metastasis (yes/no)	1.590	0.664-3.806	0.298			
Brain metastasis (yes/no)	1.528	0.209-11.156	0.676			
Sum of diameters (<median/>median)	1.039	0.566-1.907	0.901			
Tissue TMB at baseline (<75%/>75%)	1.272	0.681-2.376	0.451			
Blood TMB at baseline (<75%/>75%)	0.829	0.447-1.537	0.551			
On-treatment blood TMB (<75%/>75%)	0.144	0.074-0.281	<0.001	0.152	0.075-0.308	<0.001

HR: hazard ratio; CI: confidence interval; PS: performance score; TMB, tumor mutational burden

### On-treatment bTMB dynamics showed complementary value for predicting immunotherapy plus chemotherapy benefit

**Fig. 3:**
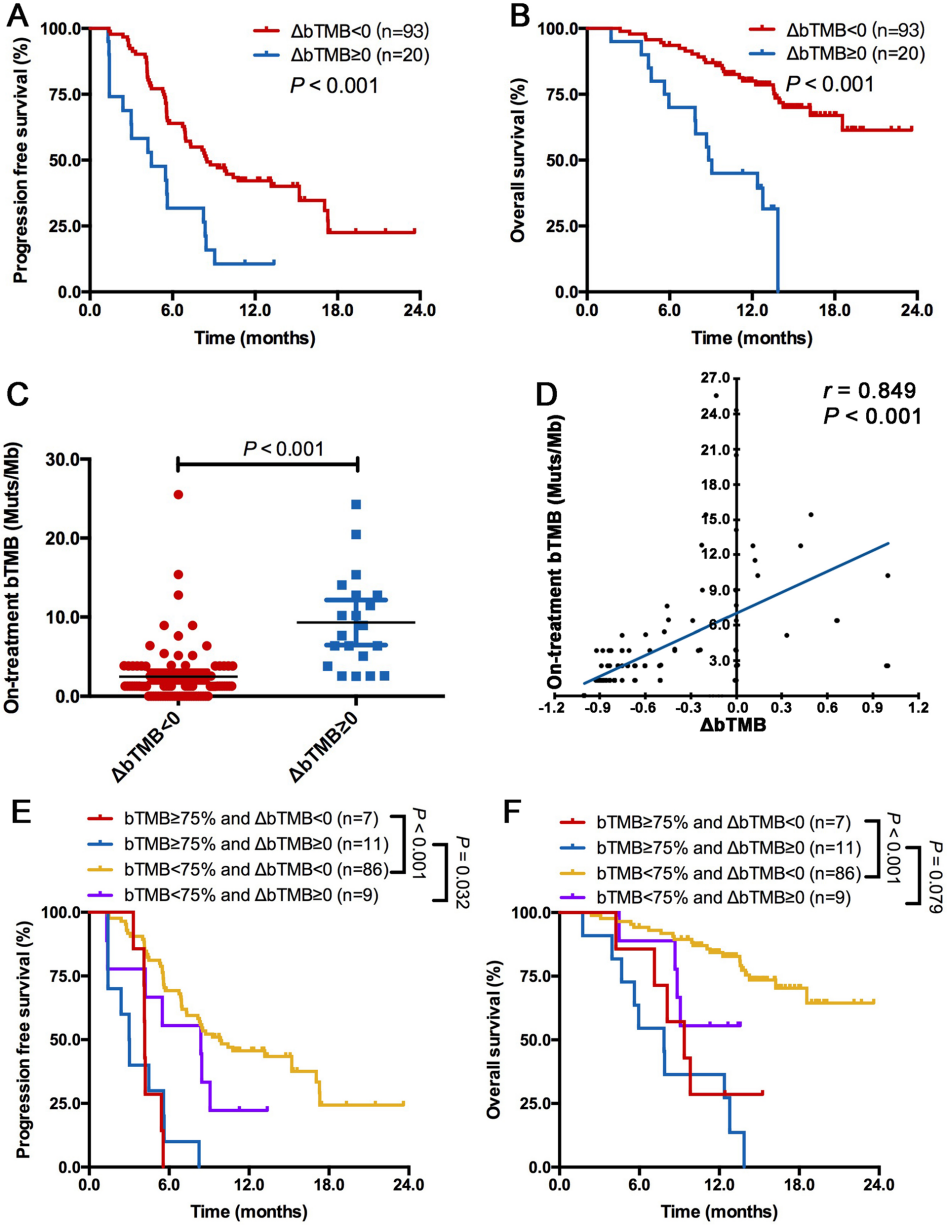
On-treatment bTMB dynamics showed complementary value for predicting immunotherapy plus chemotherapy benefit. Patients with ∆bTMB ≥0 had significantly shorter PFS (**A**) and OS (**B**) than those with ∆bTMB <0. (**C**) patients with ∆bTMB ≥0 had higher on-treatment bTMB than those with ∆bTMB <0. (D) ∆bTMB was correlated with on-treatment bTMB. Combination of on-treatment bTMB and ∆bTMB divided patients into three groups with distinct clinical outcomes: patients with low on-treatment bTMB and ∆bTMB <0 had the longest PFS (**E**) and OS (**F**), those with low on-treatment bTMB and ∆bTMB <0 or ∆bTMB ≥0 had intermediate PFS (**E**) and OS (**F**), and those with high on-treatment bTMB and ∆bTMB ≥0 had the worst PFS (**E**) and OS (**F**)

Next, we surveyed the association between bTMB dynamics (∆bTMB) and survival benefit. Patients with an increase or unchanged level in bTMB from pretreatment (defined as ∆bTMB ≥0) had obviously shorter PFS (median, 4.5 vs 8.5 months; HR = 2.545, *P* < 0.001; Figure 3A) and OS (median, 9.0 months vs not reached; HR = 4.199, *P* = 0.201; Figure 3B) than those with a decreased bTMB (defined as ∆bTMB <0). Patients with ∆bTMB ≥0 had higher on-treatment bTMB (*P* < 0.001, Figure 3C) than those with ∆bTMB <0. ∆bTMB was correlated with on-treatment bTMB (*r*^*Spearman*^= 0.849, *P* < 0.001, Figure 3D). When we combined ∆bTMB with on-treatment bTMB, we found that they were complementary, nonredundant correlates of treatment benefit; patients with low on-treatment bTMB and ∆bTMB <0 had the longest PFS and OS, those with low on-treatment bTMB and ∆bTMB <0 or ∆bTMB ≥0 had intermediate PFS and OS, and those with high on-treatment bTMB and ∆bTMB ≥0 had the worst PFS and OS (*P* < 0.001; Figure 3E and F).

### On-treatment bTMB identifies long-term benefit among patients with initially radiological SD

**Fig. 4:**
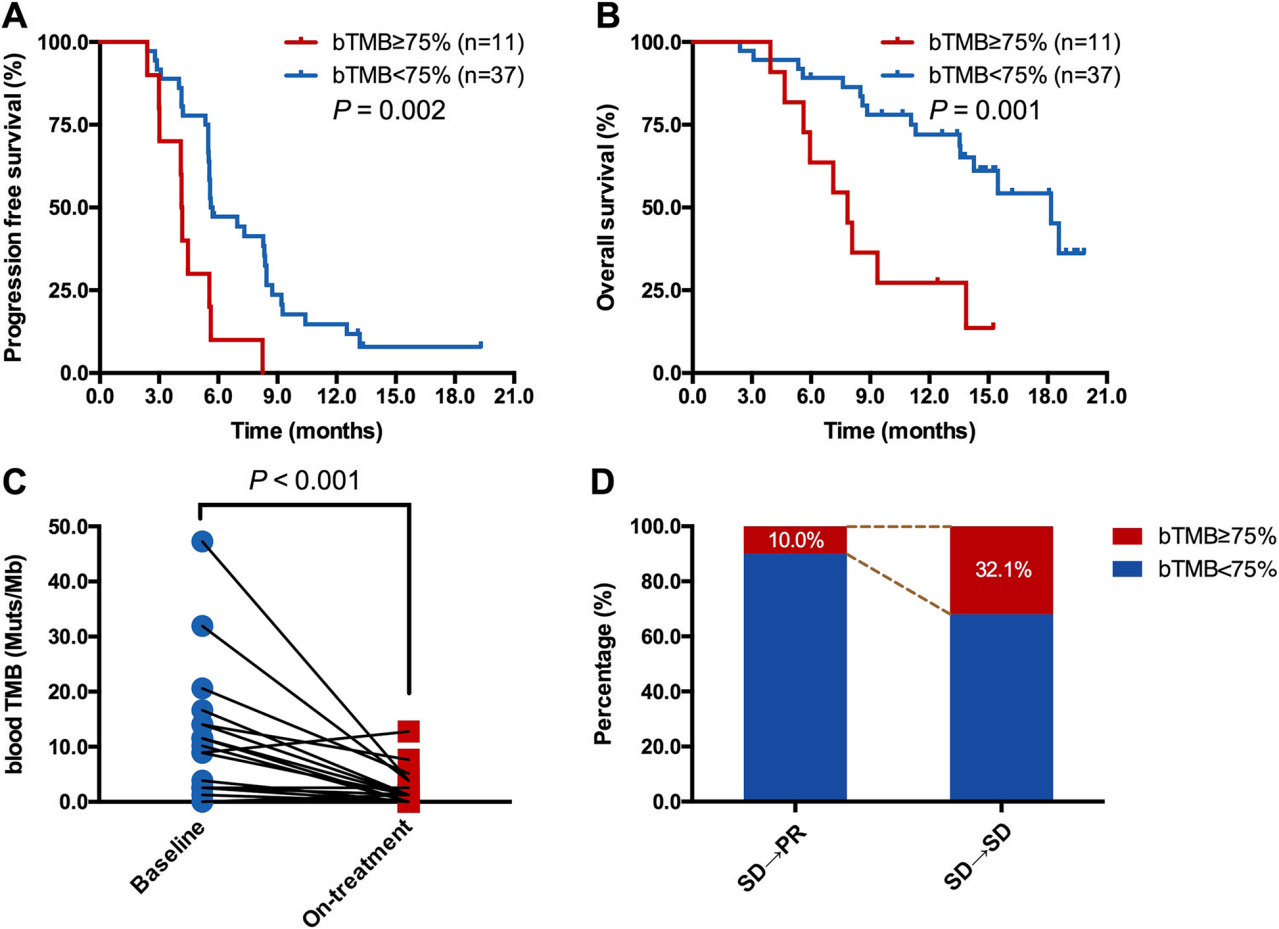
On-treatment bTMB identifies long-term benefit among patients with initially radiological SD. In patients with initially radiological SD in camrelizumab plus chemotherapy group, high on-treatment bTMB was associated with inferior PFS (**A**) and OS (**B**). (**C**) Patients who had initially radiological SD but best response of PR, had markedly reduction of bTMB after two cycles treatment. **(D)** Patients with initially radiological SD but best response of PR had lower percentage of on-treatment bTMB≥75% than those with initially radiological SD and best response of SD.

Currently, it is still a challenge to discriminate patients with initially radiologic SD would have long-term benefit from immunotherapy. Considering the correlation between on-treatment bTMB and tumor burden, we further investigated the role of on-treatment bTMB to identify patients with initially SD and would ultimately benefit from treatment. Among 48 patients with initially SD in camrelizumab plus chemotherapy group, high on-treatment bTMB was associated with significantly inferior PFS (median, 4.1 vs 5.6 months; HR = 2.861, *P* = 0.002; Figure 4A) and OS (median, 7.8 vs 18.2 months; HR = 3.546, *P* = 0.001; Figure 4B) than those with low on-treatment bTMB. Among 20 patients who had initially radiological SD and best response of PR, their on-treatment bTMB was markedly lower than the baseline bTMB (*P* < 0.001; Figure 4C). Patients with initially SD but best response of PR had lower percentage of on-treatment bTMB≥75% than those with best response of SD (10.0% vs 32.1%; Figure 4D).

## Discussion

Combination of immunotherapy and chemotherapy has become standard of care for first-line setting in advanced LUSC [6–9]. However, the predictive biomarkers for this regimen remain undetermined. Previous studies on PD-L1 expression and tTMB failed to demonstrate their predictive values [31]. In this prospective biomarker analysis from the phase III CameL-sq trial, we found that pretreatment bTMB was neither predictive nor prognostic for patients treated with camrelizumab or placebo plus chemotherapy groups. However, low on-treatment bTMB was associated with significantly better objective response, PFS and OS in camrelizumab plus chemotherapy group whereas it did not correlate with objective response and PFS in placebo plus chemotherapy group. Importantly, on-treatment bTMB dynamics showed the complementary, nonredundant value for predicting camrelizumab plus chemotherapy benefit. Furthermore, on-treatment bTMB level showed the feasibility to discriminate patients with initially radiological SD who would long-term benefit from camrelizumab plus chemotherapy. Collectively, these findings demonstrate that on-treatment bTMB together with its dynamics can serve as a predictive biomarker of immunotherapy plus chemotherapy in advanced LUSC.

Biologically, tumors with high tTMB would be inclined to have increased neoantigen production and immunogenicit y[32, 33]. tTMB has been extensively studied as a predictor for immunotherapy across tumor type s[15, 34] and found it can be utilized as a candidate biomarker of clinical outcomes from ICI-based therapy in various solid tumor s[13–15]. However, it is still a challenge for a substantial part of patients to provide sufficient tumor tissue for TMB detection. Alternatively, blood-based assay to measure TMB in plasm by ctDNA sequencing, named bTMB, was adopte d[16, 35]. Gandara et al. firstly reported that bTMB correlates with significant longer PFS in NSCLC patients received atezolizumab from combination analysis of POPLAR and OAK tria l[17]. Wang et al. showed that modified bTMB could identify patients who derive clinically significant improvements in PFS from anti–PD-(L)1 monotherapy in advanced NSCL C[18]. More recently, two retrospective studies found that bTMB can serve as a potential biomarker for predicting the efficacy of ICIs alone in NSCL C[36, 37]. However, whether bTMB could serve as a predictor for immunotherapy plus chemotherapy remains unknown. This study firstly showed that on-treatment bTMB, instead of pretreatment bTMB or tTMB, was associated with markedly superior objective response, PFS and OS in camrelizumab plus chemotherapy group while it did not correlate with objective response and PFS in placebo plus chemotherapy group, suggesting that on-treatment bTMB would be served as a feasible biomarker for camrelizumab plus chemotherapy in advanced LUSC.

Importantly, our results further demonstrated that on-treatment bTMB dynamics showed complementary, nonredundant correlates of treatment benefit. Combination of on-treatment bTMB and ∆bTMB, patients could be divided into three distinct subpopulation: patients with low on-treatment bTMB and ∆bTMB <0 had the longest PFS and OS, those with low on-treatment bTMB and ∆bTMB <0 or ∆bTMB ≥0 had intermediate PFS and OS, and the remaining had the worst PFS and OS. Similarly, a previous study reported that integration of the mean change in variant allele frequency (delta-VAF) and on-treatment VAF showed complementary correlates of prolonged survival in almost 1,000 patients treated with ICIs [38]. These results together highlight the importance of on-treatment ctDNA dynamic analysis, aiding the on-treatment bTMB calculation, to predict the benefit from immunotherapy plus chemotherapy.

Determining whether a patient with initially radiologic SD could achieve long-term benefit from immunotherapy is still a challenge in clinical setting [39]. Actually, patients with radiologic SD are a heterogeneous population including those with true response, indolent nonresponding disease, and slowly progressive disease [30, 38, 40]. Here, we firstly observed that patients with low on-treatment bTMB had a dramatically longer PFS and OS than those with high on-treatment bTMB, indicating that on-treatment bTMB could also help to adjudicate patients who would long-term benefit from camrelizumab plus chemotherapy. This finding might be vital in aiding clinical decision of continuation or early transition of treatment in patients with radiological SD, if validated in a prospective large-scale study.

There are several limitations in this study. First, the BEC population accounted for approximately 70% of the ITT population. Despite of the analogous baseline characteristics and clinical outcomes between these two groups, selection bias could be inevitable. Second, given the limited sample size, we did not further divide the BEC population into a training and validation set. Therefore, our findings still need additional independent dataset validation. Third, we defined the cutoff of high-bTMB/tTMB as TMB level ≥75%. Although it is not popular in the research setting, it is more helpful for us to clarify the relevant investigations due to its briefness. In fact, we have used different bTMB level as the cutoff and found that bTMB≥75% was the optimal cutoff with best predictive value. Forth, given the difficulty to obtain adequate high-quality tumor tissue samples, we cannot systemically evaluate the immune activation, T cell response or myeloid cell response via transcriptomic or T cell receptor repertoire analysis in the responders versus non-responders or between the different treatment groups at different time points. Last but not least, whether PD-L1 expression could show complementary value for predicting immunotherapy plus chemotherapy benefit warrant future investigations.

## Conclusion

In summary, our data firstly reported that on-treatment bTMB, instead of tTMB and pretreatment bTMB was predictive of benefit from camrelizumab plus chemotherapy in advanced LUSC. On-treatment bTMB dynamics showed complementary, nonredundant value for predicting camrelizumab plus chemotherapy benefit. On-treatment bTMB could also adjudicate long-term benefit among patients with initially radiological SD. Our analysis extended the understanding of the predictive value of bTMB in advanced LUSC treated with immunotherapy plus chemotherapy.

## Supplementary Information

**Additional file 1.**

## Data Availability

The data that support the findings of this study are available from the corresponding authors upon reasonable request. All requests for raw data will be reviewed by the Shanghai Pulmonary Hospital, Tongji University School of Medicine, and Jiangsu Hengrui Pharmaceuticals.

## Abbreviations

bTMB: Blood tumor mutation burden
ctDNA: Circulating tumor DNA
DCR: Disease control rate
FFPE: Formalin-fixed paraffin-embedded
HR: Hazard ratio
ICIs: Immune checkpoint inhibitors
InDels: Insertions/deletions
IRC: Independent review committee
LUSC: Lung squamous cell carcinoma
NGS: Next generation sequencing
OS: Overall survival
PD: Disease progression
PD-L1: Programmed death-ligand 1
PFS: Progression-free survival
TMB: Tumor mutation burden
TPS: Tumor proportion score
VAF: Variant allele frequency
WES: Whole-exome sequencing

## References

[1] Socinski MA, Obasaju C, Gandara D, Hirsch FR, Bonomi P, Bunn PA (2018). Current and Emergent Therapy Options for Advanced Squamous Cell Lung Cancer. J Thorac Oncol.

[2] Satpathy S, Krug K, Jean Beltran PM, Savage SR, Petralia F, Kumar-Sinha C *et al*: A proteogenomic portrait of lung squamous cell carcinoma. *Cell* 2021, 184:4348-4371 e4340. doi: 10.1016/j.cell.2021.07.016 10.1016/j.cell.2021.07.016 PMC8475722 34358469

[3] Cancer Genome Atlas Research N: Comprehensive genomic characterization of squamous cell lung cancers. *Nature* 2012, 489:519-525. doi: 10.1038/nature11404 10.1038/nature11404 PMC3466113 22960745

[4] Grant MJ, Herbst RS, Goldberg SB. Selecting the optimal immunotherapy regimen in driver-negative metastatic NSCLC. *Nat Rev Clin Oncol*. 2021. 10.1038/s41571-021-00520-1. 10.1038/s41571-021-00520-1 34168333

[5] Wang M, Herbst RS, Boshoff C (2021). Toward personalized treatment approaches for non-small-cell lung cancer. Nat Med.

[6] Paz-Ares L, Luft A, Vicente D, Tafreshi A, Gumus M, Mazieres J (2018). Pembrolizumab plus Chemotherapy for Squamous Non-Small-Cell Lung Cancer. N Engl J Med.

[7] Paz-Ares L, Vicente D, Tafreshi A, Robinson A, Soto Parra H, Mazieres J (2020). A Randomized, Placebo-Controlled Trial of Pembrolizumab Plus Chemotherapy in Patients With Metastatic Squamous NSCLC: Protocol-Specified Final Analysis of KEYNOTE-407. J Thorac Oncol.

[8] Wang J, Lu S, Yu X, Hu Y, Sun Y, Wang Z (2021). Tislelizumab Plus Chemotherapy vs Chemotherapy Alone as First-line Treatment for Advanced Squamous Non-Small-Cell Lung Cancer: A Phase 3 Randomized Clinical Trial. JAMA Oncol.

[9] Zhou C, Wu L, Fan Y, Wang Z, Liu L, Chen G, et al. Sintilimab Plus Platinum and Gemcitabine as First-Line Treatment for Advanced or Metastatic Squamous NSCLC: Results From a Randomized, Double-Blind, Phase 3 Trial (ORIENT-12). *J Thorac Oncol*. 2021. 10.1016/j.jtho.2021.04.011. 10.1016/j.jtho.2021.04.011 34048947

[10] Rizvi NA, Hellmann MD, Snyder A, Kvistborg P, Makarov V, Havel JJ, et al. Cancer immunology. Mutational landscape determines sensitivity to PD-1 blockade in non-small cell lung cancer. *Science*. 2015;348(124-128). 10.1126/science.aaa1348. 10.1126/science.aaa1348 PMC4993154 25765070

[11] Yarchoan M, Hopkins A, Jaffee EM (2017). Tumor Mutational Burden and Response Rate to PD-1 Inhibition. N Engl J Med.

[12] Samstein RM, Lee CH, Shoushtari AN, Hellmann MD, Shen R, Janjigian YY (2019). Tumor mutational load predicts survival after immunotherapy across multiple cancer types. Nat Genet.

[13] Hellmann MD, Callahan MK, Awad MM, Calvo E, Ascierto PA, Atmaca A (2018). Tumor Mutational Burden and Efficacy of Nivolumab Monotherapy and in Combination with Ipilimumab in Small-Cell Lung Cancer. Cancer Cell.

[14] Hellmann MD, Ciuleanu TE, Pluzanski A, Lee JS, Otterson GA, Audigier-Valette C (2018). Nivolumab plus Ipilimumab in Lung Cancer with a High Tumor Mutational Burden. N Engl J Med.

[15] Marabelle A, Fakih M, Lopez J, Shah M, Shapira-Frommer R, Nakagawa K (2020). Association of tumour mutational burden with outcomes in patients with advanced solid tumours treated with pembrolizumab: prospective biomarker analysis of the multicohort, open-label, phase 2 KEYNOTE-158 study. Lancet Oncol.

[16] Chae YK, Davis AA, Agte S, Pan A, Simon NI, Iams WT (2019). Clinical Implications of Circulating Tumor DNA Tumor Mutational Burden (ctDNA TMB) in Non-Small Cell Lung Cancer. Oncologist.

[17] Gandara DR, Paul SM, Kowanetz M, Schleifman E, Zou W, Li Y (2018). Blood-based tumor mutational burden as a predictor of clinical benefit in non-small-cell lung cancer patients treated with atezolizumab. Nat Med.

[18] Wang Z, Duan J, Cai S, Han M, Dong H, Zhao J (2019). Assessment of Blood Tumor Mutational Burden as a Potential Biomarker for Immunotherapy in Patients With Non-Small Cell Lung Cancer With Use of a Next-Generation Sequencing Cancer Gene Panel. JAMA Oncol.

[19] Zhou CC RS, Chen JH, et al. : Camrelizumab or placebo plus carboplatin and paclitaxel as first-line treatment for advanced squamous NSCLC (CameL-sq): A randomized, double-blind, multicenter, phase III trial. . *ELCC 2021, abstract 96O* 2021.

[20] Bolger AM, Lohse M, Usadel B (2014). Trimmomatic: a flexible trimmer for Illumina sequence data. Bioinformatics.

[21] Li H, Handsaker B, Wysoker A, Fennell T, Ruan J, Homer N (2009). The Sequence Alignment/Map format and SAMtools. Bioinformatics.

[22] DePristo MA, Banks E, Poplin R, Garimella KV, Maguire JR, Hartl C (2011). A framework for variation discovery and genotyping using next-generation DNA sequencing data. Nat Genet.

[23] Lai Z, Markovets A, Ahdesmaki M, Chapman B, Hofmann O, McEwen R (2016). VarDict: a novel and versatile variant caller for next-generation sequencing in cancer research. Nucleic Acids Res.

[24] Wang K, Li M, Hakonarson H (2010). ANNOVAR: functional annotation of genetic variants from high-throughput sequencing data. Nucleic Acids Res.

[25] Amemiya HM, Kundaje A, Boyle AP (2019). The ENCODE Blacklist: Identification of Problematic Regions of the Genome. Sci Rep.

[26] van der Leest P, Hiddinga B, Miedema A, Aguirre Azpurua ML, Rifaela N, Ter Elst A (2021). Circulating tumor DNA as a biomarker for monitoring early treatment responses of patients with advanced lung adenocarcinoma receiving immune checkpoint inhibitors. Mol Oncol.

[27] Laza-Briviesca R, Cruz-Bermudez A, Nadal E, Insa A, Garcia-Campelo MDR, Huidobro G (2021). Blood biomarkers associated to complete pathological response on NSCLC patients treated with neoadjuvant chemoimmunotherapy included in NADIM clinical trial. Clin Transl Med.

[28] Nabet BY, Esfahani MS, Moding EJ, Hamilton EG, Chabon JJ, Rizvi H (2020). Noninvasive Early Identification of Therapeutic Benefit from Immune Checkpoint Inhibition. Cell.

[29] Raja R, Kuziora M, Brohawn PZ, Higgs BW, Gupta A, Dennis PA (2018). Early Reduction in ctDNA Predicts Survival in Patients with Lung and Bladder Cancer Treated with Durvalumab. Clin Cancer Res.

[30] Goldberg SB, Narayan A, Kole AJ, Decker RH, Teysir J, Carriero NJ (2018). Early Assessment of Lung Cancer Immunotherapy Response via Circulating Tumor DNA. Clin Cancer Res.

[31] Gadgeel S, Rodriguez-Abreu D, Speranza G, Esteban E, Felip E, Domine M (2020). Updated Analysis From KEYNOTE-189: Pembrolizumab or Placebo Plus Pemetrexed and Platinum for Previously Untreated Metastatic Nonsquamous Non-Small-Cell Lung Cancer. J Clin Oncol.

[32] Schumacher TN, Schreiber RD (2015). Neoantigens in cancer immunotherapy. Science.

[33] Galluzzi L, Chan TA, Kroemer G, Wolchok JD, Lopez-Soto A. The hallmarks of successful anticancer immunotherapy. *Sci Transl Med*. 2018;10. 10.1126/scitranslmed.aat7807. 10.1126/scitranslmed.aat7807 30232229

[34] Chan TA, Yarchoan M, Jaffee E, Swanton C, Quezada SA, Stenzinger A (2019). Development of tumor mutation burden as an immunotherapy biomarker: utility for the oncology clinic. Ann Oncol.

[35] Killock D (2019). bTMB is a promising predictive biomarker. Nat Rev Clin Oncol.

[36] Chen X, Fang L, Zhu Y, Bao Z, Wang Q, Liu R, et al. Blood tumor mutation burden can predict the clinical response to immune checkpoint inhibitors in advanced non-small cell lung cancer patients. *Cancer Immunol Immunother*. 2021. 10.1007/s00262-021-02943-2. 10.1007/s00262-021-02943-2 PMC10991091 33899131

[37] Ma Y, Li Q, Du Y, Cai J, Chen W, Zhao G (2021). Blood Tumor Mutational Burden as a Predictive Biomarker in Patients With Advanced Non-Small Cell Lung Cancer (NSCLC). Front Oncol.

[38] Zhang Q, Luo J, Wu S, Si H, Gao C, Xu W (2020). Prognostic and Predictive Impact of Circulating Tumor DNA in Patients with Advanced Cancers Treated with Immune Checkpoint Blockade. Cancer Discov.

[39] Hegde PS, Chen DS (2020). Top 10 Challenges in Cancer Immunotherapy. Immunity.

[40] Anagnostou V, Forde PM, White JR, Niknafs N, Hruban C, Naidoo J (2019). Dynamics of Tumor and Immune Responses during Immune Checkpoint Blockade in Non-Small Cell Lung Cancer. Cancer Res.

