# Supplementary material for: On-treatment blood TMB as predictors for camrelizumab plus chemotherapy in advanced lung squamous cell carcinoma: biomarker analysis of a phase III trial
Source: Mol Cancer. 2022 Jan 3;21:4. doi: 10.1186/s12943-021-01479-4 (PMC8722280; doi:10.1186/s12943-021-01479-4)
Supplement: Supplementary file 1 — Additional file 1. [file 12943_2021_1479_MOESM1_ESM.docx]

**Supplemental Materials**

**On-treatment blood TMB as predictors for camrelizumab plus chemotherapy in advanced lung squamous cell carcinoma: biomarker analysis of a phase III trial**

**Supplemental Table S1........................................................................Page 2.**

**Supplemental Figure S1......................................................................Page 3.**

**Supplemental Figure S2......................................................................Page 4.**

**Supplemental Figure S3......................................................................Page 5.**

**Supplemental Figure S4......................................................................Page 6.**

**Supplemental Figure S5......................................................................Page 7.**

**Supplemental Figure S6......................................................................Page 8.**

**Supplemental Figure S7......................................................................Page 9.**

## Table S1: Baseline characteristics of included patients.

|  | **Camrelizumb plus chemotherapy** | **Placebo plus chemotherapy** |
| --- | --- | --- |
|  | **(n=134)** | **(n=136)** |
| Age |  |  |
| Median (range), years | 64 (34-74) | 63 (36-73) |
| ≥65 years | 79 (59%) | 92 (68%) |
| <65 years | 55 (41%) | 44 (32%) |
| Sex |  |  |
| Male | 128 (96%) | 128 (94%) |
| Female | 6 (4%) | 8 (6%) |
| Smoking history |  |  |
| ≥400 cigarette-years | 117 (87%) | 112 (82%) |
| <400 cigarette-years | 6 (4%) | 11 (8%) |
| Never | 11 (9%) | 13 (10%) |
| ECOG performance status | | |
| 0 | 25 (19%) | 30 (22%) |
| 1 | 109 (81%) | 106 (78%) |
| Disease stage |  |  |
| IIIB/IIIC | 40 (30%) | 40 (29%) |
| IV | 94 (70%) | 96 (71%) |
| Liver or brain metastases at enrollment^*^ | 0 (0%) | 0 (0%) |
| Liver metastases | 14 (10%) | 13 (10%) |
| Brain metastases | 2 (1%) | 3 (1%) |
| PD-L1 tumor proportion score | | |
| <1% | 61 (46%) | 68 (50%) |
| ≥1% | 70 (52%) | 67 (49%) |
| 1-49% | 36 (27%) | 39 (29%) |
| ≥50% | 34 (25%) | 28 (21%) |
| Not evaluable | 3 (2%) | 1 (1%) |

Data are n (%), unless otherwise indicated. ^*^ No patients with both liver and lung metastases were enrolled. ECOG, Eastern Cooperative Oncology Group.


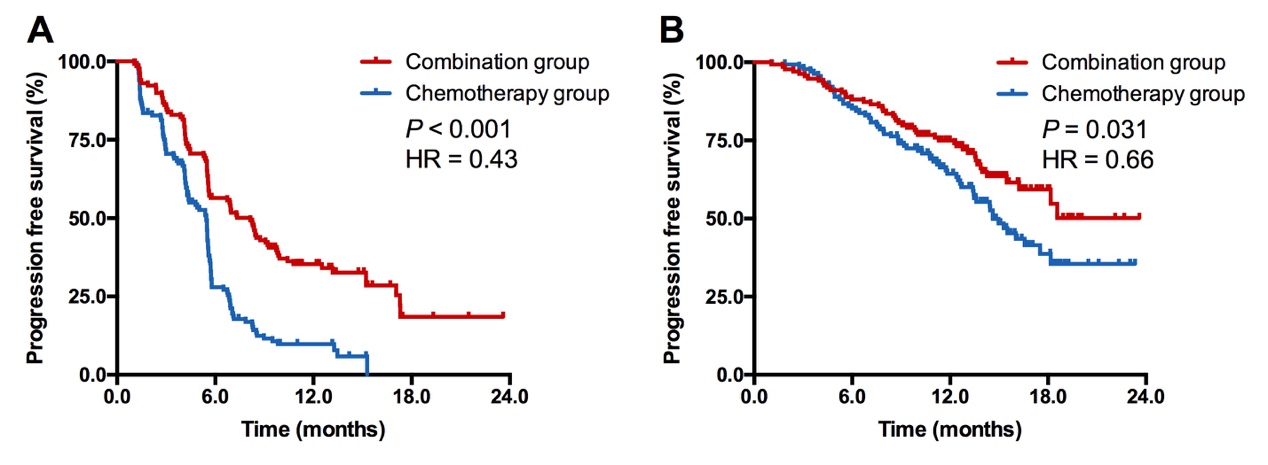


**Supplemental Figure S1.** **Progression-free (A) and overall survival (B) in biomarker evaluable cohort. HR, hazard ratio.**

**
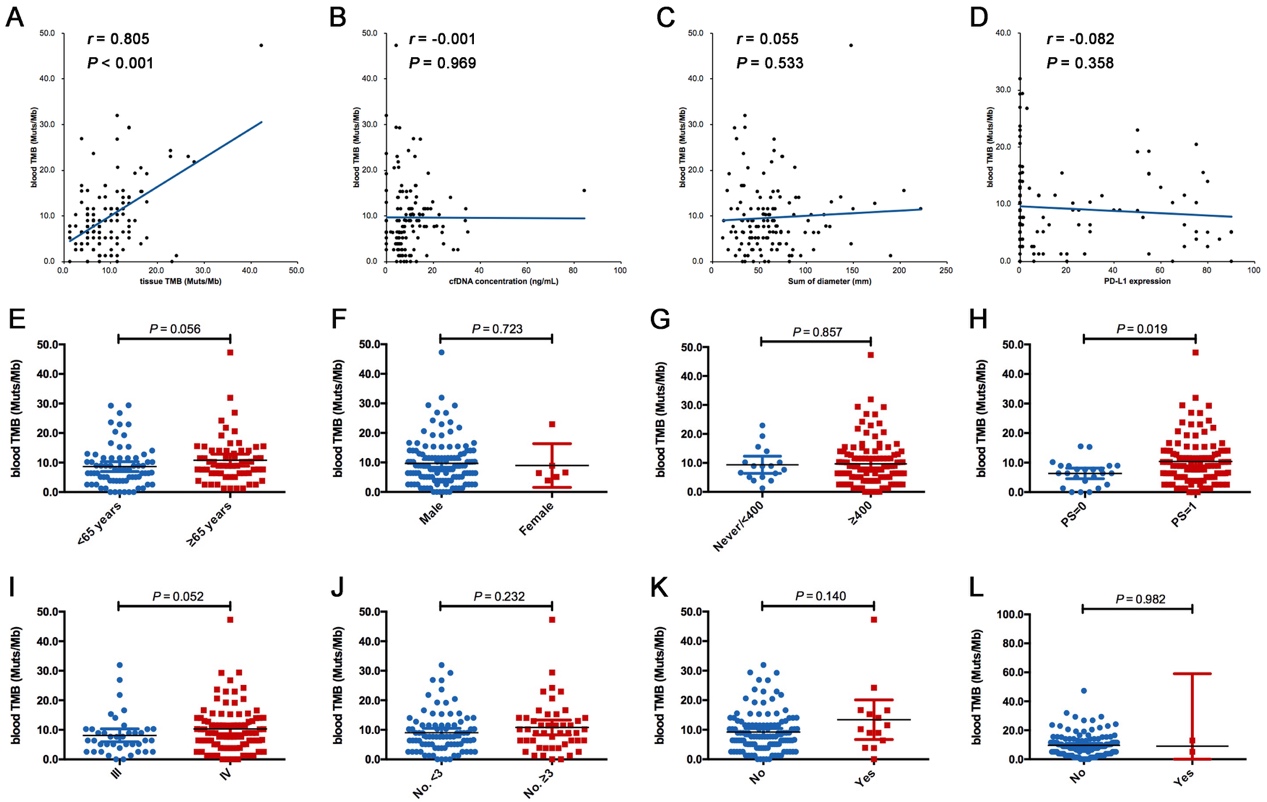
**

**Supplemental Figure S2.** **Correlation between baseline clinicopathological features and pretreatment bTMB.** (A) Correlation between tTMB and pretreatment bTMB; (B) Correlation between cfDNA concentration and pretreatment bTMB; (C) Correlation between sum of diameter of target lesions and pretreatment bTMB; (D) Correlation between PD-L1 expression and pretreatment bTMB; (E) Correlation between age and pretreatment bTMB; (F) Correlation between sex and pretreatment bTMB; (G) Correlation between smoking history and pretreatment bTMB; (H) Correlation between ECOG PS and pretreatment bTMB; (I) Correlation between disease stage and pretreatment bTMB; (J) Correlation between number of distant metastasis and pretreatment bTMB; (K) Correlation between liver metastasis and pretreatment bTMB (Yes, with liver metastasis; No, without liver metastasis); (L) Correlation between brain metastasis and pretreatment bTMB (Yes, with brain metastasis; No, without brain metastasis).

**
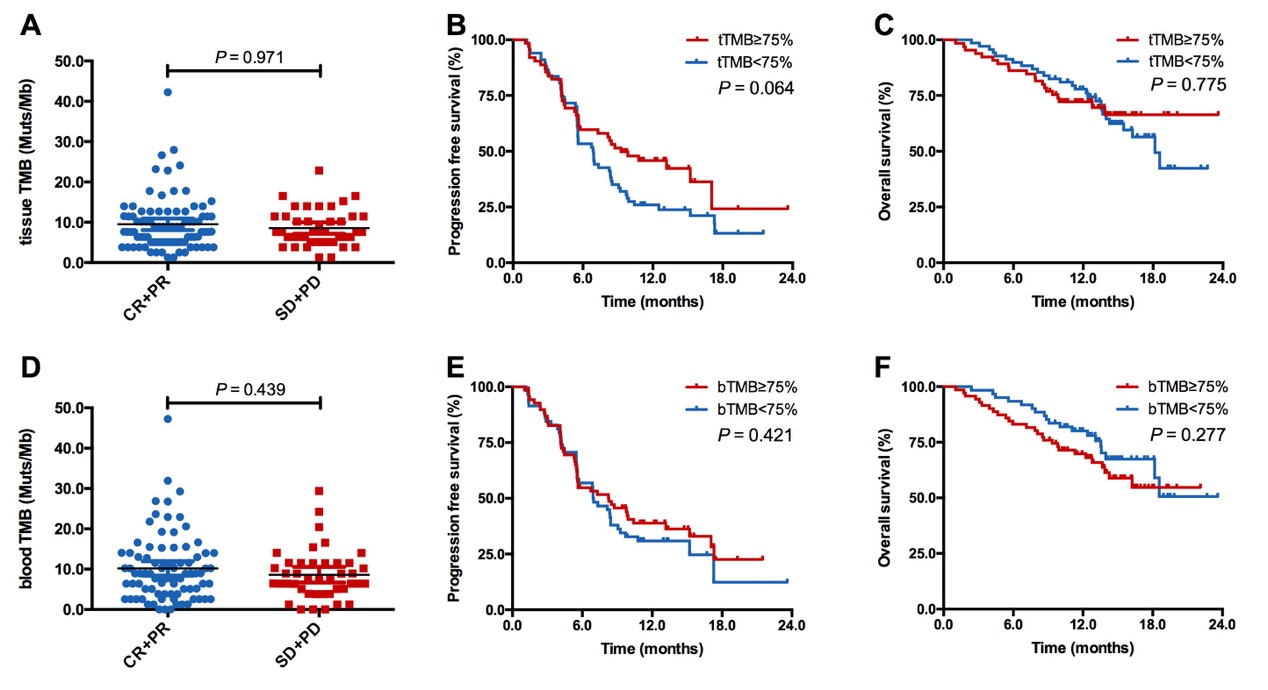
**

**Supplemental Figure S3.** **Predictive and prognostic value of pretreatment bTMB and tTMB in camrelizumab plus chemotherapy group.** (A-C) Pretreatment tTMB did not correlated with ORR (A), PFS (B) and OS (C). (D-F) Pretreatment bTMB did not correlated with ORR (D), PFS (E) and OS (F).

**
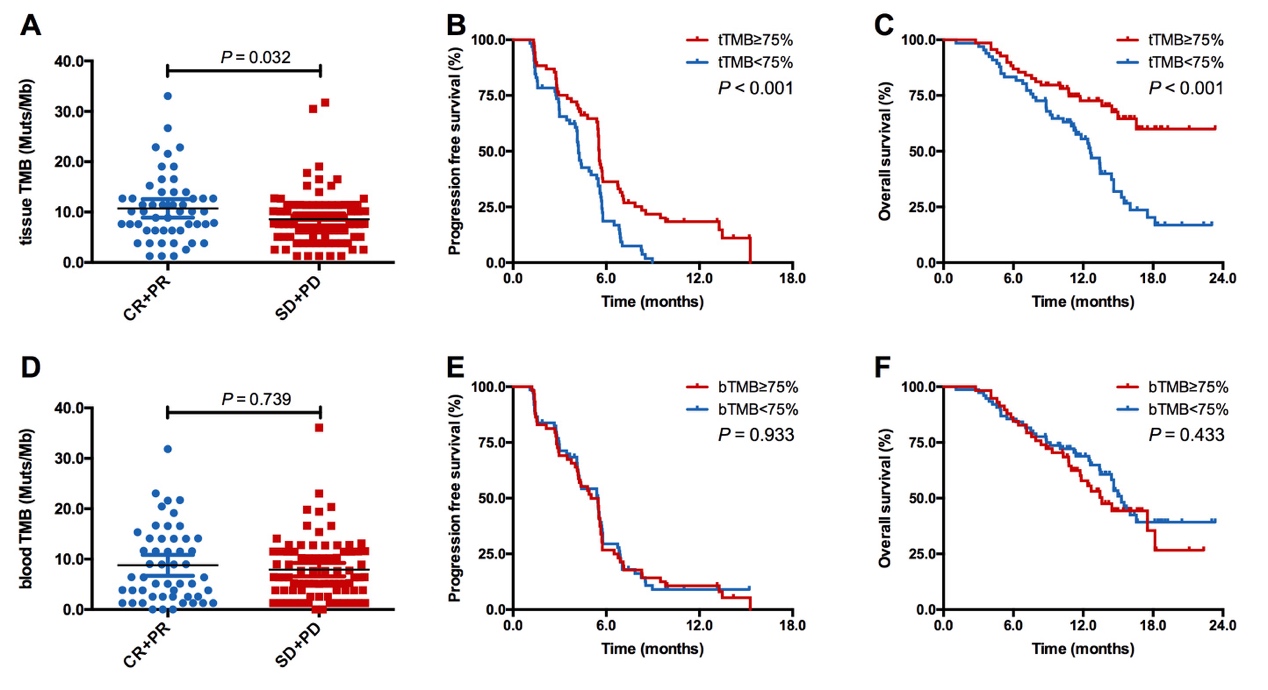
**

**Supplemental Figure S4.** **Predictive and prognostic value of pretreatment bTMB and tTMB in placebo plus chemotherapy group.** (A-C) Pretreatment tTMB did not correlated with ORR (A), PFS (B) and OS (C). (D-F) Pretreatment bTMB did not correlated with ORR (D), PFS (E) and OS (F).

**
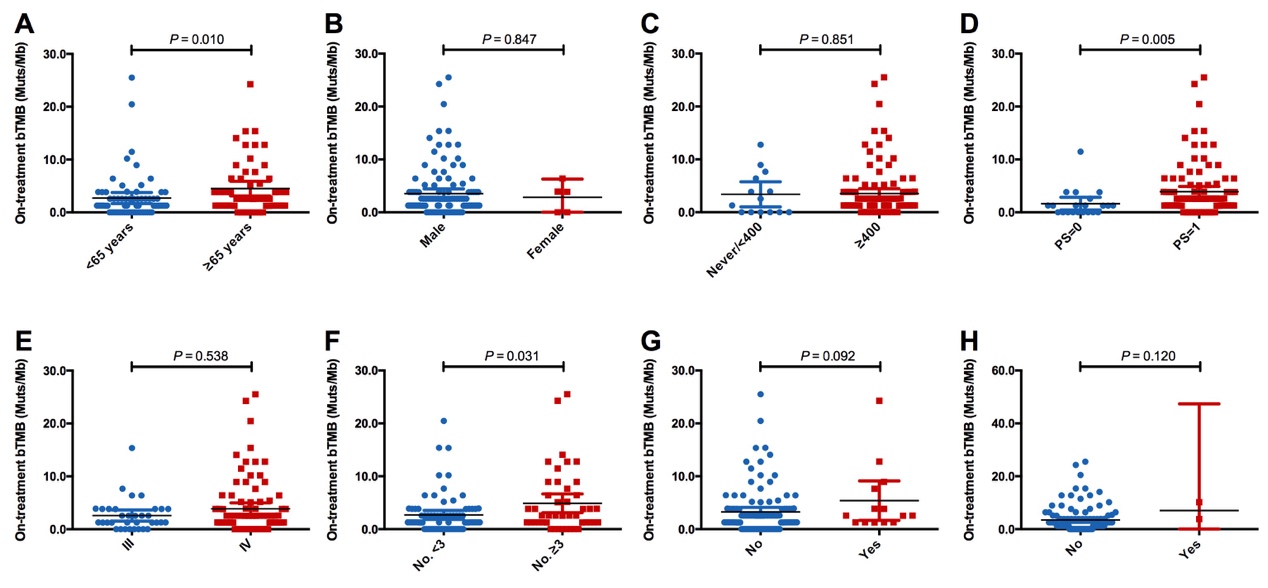
**

**Supplemental Figure S5.** **Correlation between baseline clinicopathological features and on-treatment bTMB.** (A) Correlation between age and on-treatment bTMB; (B) Correlation between sex and on-treatment bTMB; (C) Correlation between smoking history and on-treatment bTMB; (D) Correlation between ECOG PS and on-treatment bTMB; (E) Correlation between disease stage and on-treatment bTMB; (F) Correlation between number of distant metastasis and on-treatment bTMB; (G) Correlation between liver metastasis and on-treatment bTMB (Yes, with liver metastasis; No, without liver metastasis); (H) Correlation between brain metastasis and on-treatment bTMB (Yes, with brain metastasis; No, without brain metastasis).

**
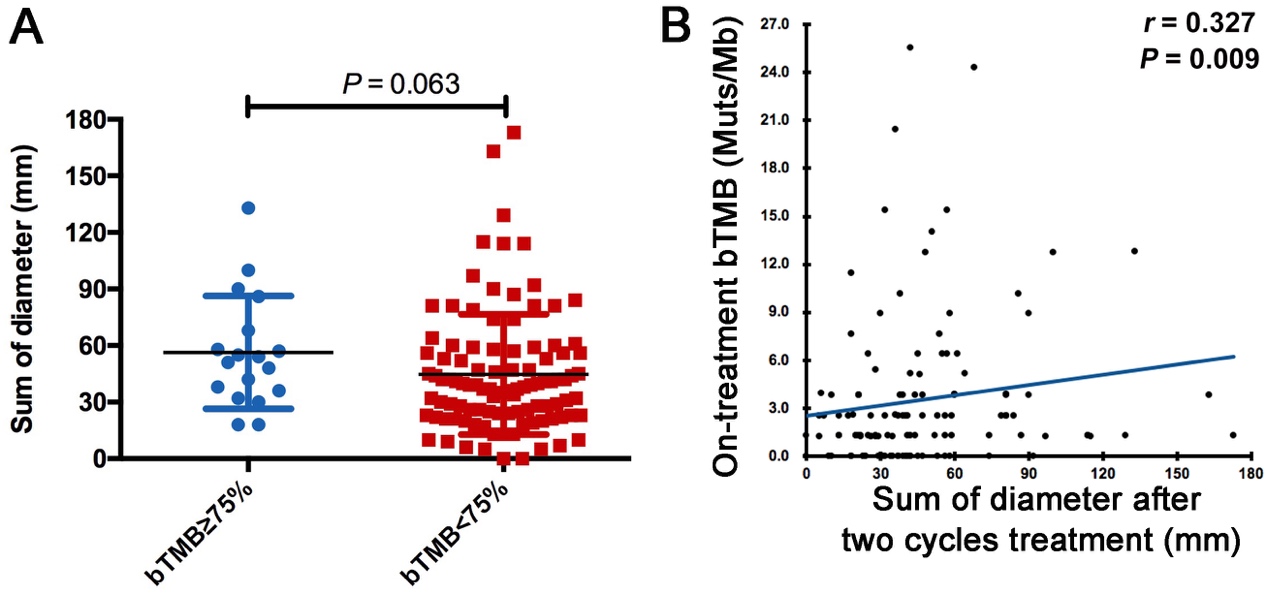
**

**Supplemental Figure S6.** **Correlation between sum of diameters of target lesions after two cycles treatment and on-treatment bTMB.** Sum of diameters of target lesions is a sum of the diameters (longest for non-nodal lesions, short axis for nodal lesions) of target lesions will be calculated and reported as the baseline sum diameters according to Response Evaluation Criteria in Solid Tumors (RECIST) version 1.1. (A) Patients with high on-treatment bTMB had longer sum of the diameters of the target lesions after two cycles treatment; (B) On-treatment bTMB level also correlated with the sum of the diameters of the target lesions after two cycles treatment.

**
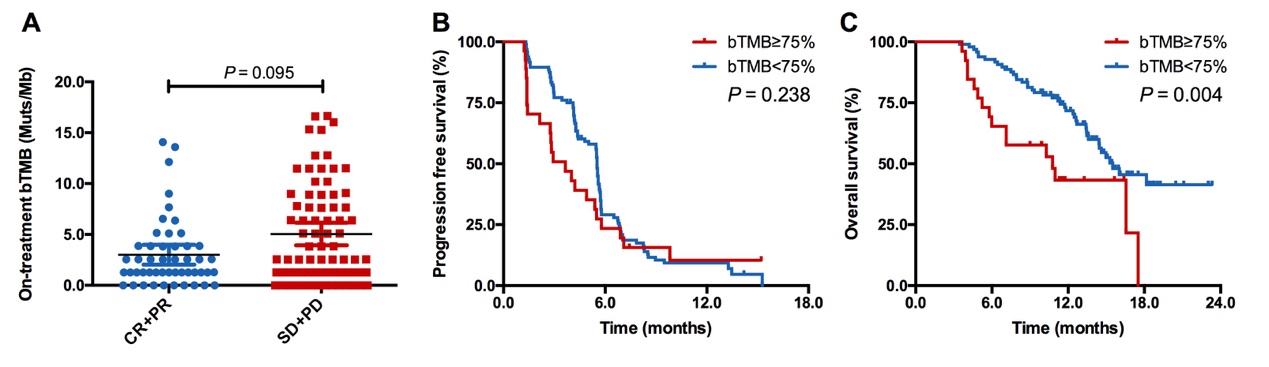
**

**Supplemental Figure S7.** **Predictive and prognostic value of on-treatment bTMB in placebo plus chemotherapy group.** (A-C) On-treatment bTMB did not correlate with ORR (A) and PFS (B) while it was only associated with OS (C).
